# Dual Short Upstream Open Reading Frames Control Translation of a Herpesviral Polycistronic mRNA

**DOI:** 10.1371/journal.ppat.1003156

**Published:** 2013-01-31

**Authors:** Lisa M. Kronstad, Kevin F. Brulois, Jae U. Jung, Britt A. Glaunsinger

**Affiliations:** 1 Department of Plant and Microbial Biology, University of California, Berkeley, Berkeley, California, United States of America; 2 Department of Molecular Microbiology and Immunology, Keck School of Medicine, University of California, Los Angeles, Los Angeles, California, United States of America; University of North Carolina, United States of America

## Abstract

The Kaposi's sarcoma-associated herpesvirus (KSHV) protein kinase, encoded by ORF36, functions to phosphorylate cellular and viral targets important in the KSHV lifecycle and to activate the anti-viral prodrug ganciclovir. Unlike the vast majority of mapped KSHV genes, no viral transcript has been identified with ORF36 positioned as the 5′-proximal gene. Here we report that ORF36 is robustly translated as a downstream cistron from the ORF35–37 polycistronic transcript in a cap-dependent manner. We identified two short, upstream open reading frames (uORFs) within the 5′ UTR of the polycistronic mRNA. While both uORFs function as negative regulators of ORF35, unexpectedly, the second allows for the translation of the downstream ORF36 gene by a termination-reinitiation mechanism. Positional conservation of uORFs within a number of related viruses suggests that this may be a common γ-herpesviral adaptation of a host translational regulatory mechanism.

## Author Summary

Kaposi's sarcoma-associated herpesvirus (KSHV) is the etiologic agent of multicentric Castleman's disease, primary effusion lymphoma and Kaposi's sarcoma. KSHV expresses a number of transcripts with the potential to generate multiple proteins, yet relies on the cellular translation machinery that is primed to synthesize only one protein per mRNA. Here we report that the viral transcript encompassing ORF35–37 is able to direct synthesis of two proteins and that the translational switch is regulated by two short upstream open reading frames (uORFs) in the native 5′ untranslated region. uORFs are elements commonly found upstream of mammalian genes that function to interfere with unrestrained ribosomal scanning and thus repress translation of the major ORF. The sequence of the viral uORF appears unimportant, and instead functions to position the translation machinery in a location that favors translation of the downstream major ORF, via a reinitiation mechanism. Thus, KSHV uses a host strategy generally reserved to repress translation to instead allow for the expression of an internal gene.

## Introduction

Translation initiation of eukaryotic mRNAs is dependent on the 5′ mRNA cap and proceeds by ribosomal scanning until recognition of an AUG codon in a favorable context [1], [2]. As a consequence of the translation machinery not engaging start codons at internal positions within the mRNA, eukaryotic transcripts generally encode only one functional protein. For the majority of mRNAs the most 5′-proximal AUG is selected, however strategies exist to bypass upstream start codons to enable downstream initiation. For example, leaky scanning can occur if the nucleotides flanking the 5′-proximal AUG are not in the Kozak consensus sequence (ccRccAUGG), allowing the 40S ribosomal subunit to instead engage a downstream methionine codon [2], [3]. Alternatively, when an upstream AUG is followed shortly thereafter by an in-frame termination codon, ribosomes can reinitiate translation, albeit with reduced efficiency, at a downstream AUG. These upstream open reading frames (uORFs) presumably permit translation of a downstream gene because factors necessary for initiation have not yet dissociated during the short elongation period. Notably, uORFs are common regulatory elements in eukaryotic transcripts, and generally function to reduce translation of the major ORF [3], [4]. Additional, although rare, examples of internal ORF translation also exist, for example after ribosome shunting over a highly structured upstream sequence [5]–[8], or upon direct 40S recruitment via internal ribosome entry sites (IRESs) [9]–[13].

Viruses do not encode translation machinery and thus operate under the constraints of host protein synthesis. However, the compact nature of viral genomes has resulted in the evolution of specialized strategies to maximize their coding capacity. Examples of such mechanisms include translation of a large polyprotein that is cleaved into multiple proteins, ribosomal frameshifting and non-canonical translation mechanisms such as those described above [14]. Accordingly, many viral mRNAs do not conform to the one protein per mRNA cellular paradigm and require specialized mechanisms to subvert the translational constraints of the host.

Kaposi's sarcoma-associated herpesvirus (KSHV) is the etiologic agent of several human cancers including multicentric Castleman's disease, primary effusion lymphoma and Kaposi's sarcoma (KS), one of the early AIDS-defining illnesses [15]–[17]. KSHV is a double-stranded DNA virus of the γ-herpesvirus subfamily, possessing a ∼165-kb genome and encoding an estimated 80 viral proteins [17], [18]. The viral genes closely resemble those of their cellular counterparts in that they have canonical transcriptional promoters, consensus pre-mRNA splice sites and 3′-end formation signals. However, one notable departure from the cellular paradigm is the scarcity of poly(A) sites distributed throughout the genome, with a single signal often allocated to several consecutive ORFs. These gene clusters give rise to viral transcripts with polycistronic coding potential, although in general only the 5′-proximal gene is translated on each mRNA [19]–[21]. Most genes are positioned as a 5′ cistron by the use of multiple transcriptional start sites upstream of common poly(A) signals and/or alternative splicing [21], [22]. To date, the only described KSHV mechanism to enable translation of a 3′-proximal ORF is an IRES identified in the coding region of vCyclin (ORF72), which allows for expression of vFLIP (ORF71) [23]–[25].

A previously described tricistronic KSHV mRNA encompasses three partially overlapping open reading frames that are expressed with lytic kinetics (ORF35, 36, and 37). However, the mechanism of translation initiation of the 5′-distal ORF36 and ORF37 proteins has remained unresolved [26], [27]. The function of the protein product of the 5′-proximal ORF35 is ill defined, although it shares limited sequence similarity with the α-herpesvirus UL14 gene product, which has described heat shock protein-like properties and functions to inhibit apoptosis during host cell infection [28], [29]. The second gene, ORF36, encodes a serine/threonine kinase that activates the cellular c-Jun N-terminal kinase (JNK) signaling pathway and phosphorylates the viral transcriptional transactivator K-bZIP, two processes involved in the progression from early to late viral gene expression [27], [30], [31]. Moreover, ORF36 sensitizes KSHV-infected cells to ganciclovir, an anti-viral drug shown to reduce KSHV replication in cultured cells and in patients [32]–[35]. The 3′-proximal ORF37 expresses SOX (*s*hut*o*ff and e*x*onuclease), a viral protein responsible for promoting widespread degradation of host mRNAs and also thought to assist in viral DNA replication and packaging [36]–[38].

Here, we demonstrate that the ORF35–37 transcript is functionally bicistronic, supporting translation of both ORF35 and ORF36, whereas ORF37 is expressed from a previously uncharacterized monocistronic transcript. The polycistronic locus lacks IRES activity, and both proteins are expressed in a cap-dependent manner. Interestingly, translation of ORF36 occurs via a reinitiation mechanism after engagement of one of two overlapping short uORFs located in the 5′-untranslated region (UTR), which also regulate the relative expression levels of these proteins. Thus, KSHV uses a host strategy normally reserved to repress translation of the major ORF to instead permit expression of a 3′-proximal cistron on a viral polycistronic mRNA. Analysis of homologous genetic loci from additional γ-herpesviruses similarly revealed the presence of dual short upstream ORFs (uORFs), suggesting this may be a conserved mechanism of translation initiation among these viruses.

## Results

### Identification of a functionally bicistronic KSHV mRNA

**Figure 1:**
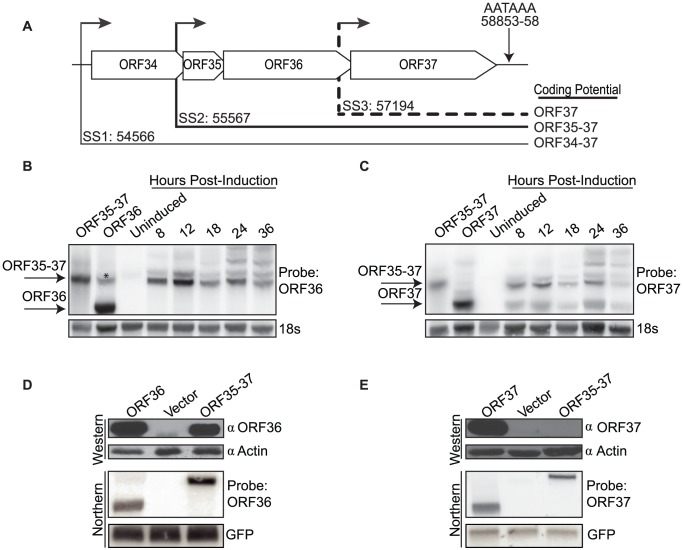
Efficient translation of ORF36, but not ORF37, occurs from the full-length ORF35–37 tricistronic transcript. (A) A schematic presentation of the *ORF34–37* genetic locus showing the previously identified ORF34–37 and ORF35–37 polycistronic mRNAs with thin and thick lines respectively. Coding potentials are indicated on the right. The ORF37-specific transcript is denoted as a dotted line. Start sites (SS) are indicated for each transcript according to the nucleotide position described by Russo et al. [18]. The single poly(A) signal used by all four ORFs for transcription termination is shown. (B–C) TREx BCBL1-RTA cells were mock treated (latent) or lytically reactivated for the indicated times. RNA was then isolated and Northern blotted with a ^32^P-labeled ORF36 (B) or ORF37 (C) strand-specific riboprobe. An additional higher molecular weight 293T-specific cross-reacting band was also detected in the ORF36 control lane, denoted by *. (D–E) 293T cells were transfected with the indicated plasmid, and total RNA and protein were isolated 24 h later. Protein lysates were resolved by SDS-PAGE and detected by Western blot with antibodies against ORF36 (D) or ORF37 (E). Actin served as a loading control. To verify transcript integrity, RNA was Northern blotted with ^32^P-labeled ORF36 (D) or ORF37 (E) DNA probes or with a probe against the GFP co-transfection control.

Two potential functionally polycistronic mRNAs are transcribed from the KSHV *ORF34–37* genetic locus during lytic replication: a minor transcript encompassing ORFs 34, 35, 36, and 37 (ORF34–37) and a major transcript encompassing ORFs 35, 36 and 37 (ORF35–37) (Figure 1A) [26], [27]. Although both ORF36 and ORF37 proteins play important roles in the viral lifecycle, no transcripts were reported in which these ORFs were present as the 5′-proximal cistron [26], [27]. To confirm this observation, we searched for transcripts produced from this locus in a B cell line (TREx BCBL1-RTA) that harbors KSHV in a latent state but can be stimulated to engage in lytic replication. RNA isolated from cells infected latently or lytically for 8–36 h was Northern blotted with riboprobes specific for ORF36 or ORF37. In infected cells, the ORF36 probe recognized transcripts co-migrating with or larger than the polycistronic ORF35–37 mRNA but did not reveal any smaller, potentially monocistronic species (Figure 1B). Results from ORF36 5′ rapid amplification of cDNA ends (RACE) experiments were in agreement with its transcript initiating upstream of ORF35 at nucleotide position 55567 as previously reported by Haque et al. (Figure 1A, data not shown) [18]. In contrast, the ORF37 probe reacted with transcripts ≥3.4 kb and an additional ∼1.7 kb transcript that co-migrated with the control ORF37 monocistronic mRNA (Figure 1C). Analysis of transcription start sites by 5′ RACE (data not shown), as well as similar observations in a related γ-herpesvirus further supported the presence of an ORF37 monocistronic transcript [39]. Thus, ORF37 is most likely translated by the canonical cap-dependent scanning mechanism and is present as a silent cistron on the ORF35–37 polycistronic mRNA.

We next sought to evaluate directly whether the ORF35–37 transcript could support translation of ORF36 as a downstream gene. 293T cells were first transfected with a plasmid expressing the coding sequence of ORF35–37 downstream of the native viral 72-nt 5′ UTR, and lysates were Western blotted using polyclonal antisera specific for ORF36 or, as a control, ORF37. The ORF36 protein was readily translated from this polycistronic construct, whereas the ORF37 protein was detected only in cells transfected with the monocistronic ORF37 plasmid (Figure 1D, 1E). In these and all subsequent experiments, Northern blotting of the mRNAs produced from each transfection confirmed that the transcripts were of the expected size and of equivalent abundance across experiments (Figure 1D, 1E).

**Figure 2:**
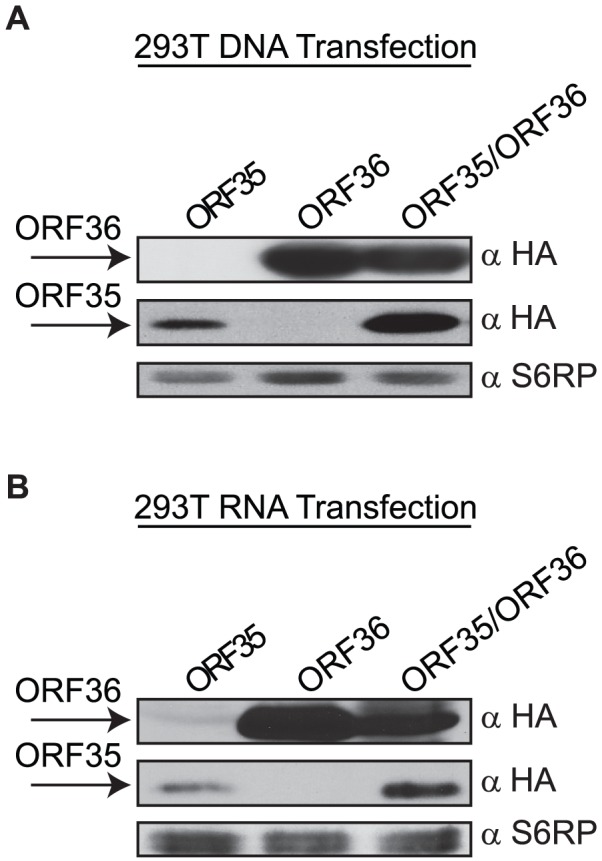
The ORF35–37 mRNA is functionally bicistronic. (A) Western blot analysis of 293T cells transfected with either N-terminally HA-tagged ORF35 with the native 5′ UTR (ORF35), C-terminally HA tagged ORF36 (ORF36) or the full length 5′ UTR HA-ORF35-ORF36-HA (ORF35/ORF36) DNA constructs. Equivalent amounts of protein lysates were resolved by SDS-PAGE and detected with anti-HA antibodies. (B) 293T cells were transfected with the indicated *in vitro* transcribed capped and polyadenylated RNA. Protein lysates were harvested 4 h post-transfection, resolved by SDS-PAGE and detected with anti-HA antibodies. The ribosomal protein S6RP served as a loading control for both experiments.

ORF35 is conserved between the α, β, and γ-herpesvirus subfamilies but its function remains unknown and antibodies are not available to detect it in KSHV-infected cells [40]. ORF35 is predicted to encode a 151-amino acid protein, and its start site resides in a favorable Kozak context. Nonetheless, we considered the possibility that ORF35 is not translated, instead serving as a portion of the 5′ UTR for ORF36. In order to directly compare the levels of ORF35 and ORF36 protein produced from the bicistronic construct, we engineered in-frame HA tags at the 5′ or 3′ end of each respective gene, maintaining the native viral 5′ UTR (5′ UTR HA-ORF35-ORF36-HA). Monocistronic versions of each HA-tagged gene were also generated as controls (5′ UTR HA-ORF35, ORF36-HA). Importantly, Western blotting with HA antibodies revealed that the ORF35 protein is produced from both the monocistronic and bicistronic constructs (Figure 2A).

Although our data indicated that the ORF35–37 transcript is functionally bicistronic, it was still formally possible that ORF36 translation occurred from a low-abundance monocistronic transcript generated by a cryptic internal promoter or splice site(s) in the DNA plasmid. To address this possibility, we transfected cells directly with *in vitro* transcribed monocistronic or bicistronic mRNAs, and performed anti-HA Western blots to detect each protein (Figure 2B). Again, both ORF35 and ORF36 protein were produced from the bicistronic 5′ UTR HA-ORF35-ORF36-HA mRNA, as well as from the appropriate control monocistronic mRNA, confirming that this locus is functionally polycistronic.

### ORF36 translation is not IRES-dependent

**Figure 3:**
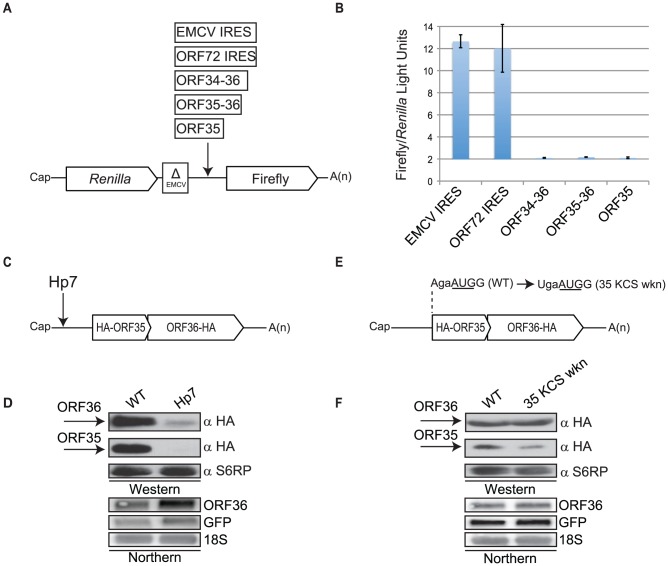
Translation of ORF36 is independent of IRES activity and dependent on the 5′ mRNA cap. (A) Diagram of dual luciferase transcripts. (B) The indicated *in vitro* transcribed, polyadenylated transcripts were electroporated into lytically reactivated TREx BCBL1-RTA cells. A dual luciferase assay was performed 4 h post-electroporation to determine the relative levels of firefly and *Renilla* luciferase activity. Experiment was performed in triplicate, error bars represent the standard deviation between replicates. (C) Schematic of 5′ UTR HA-ORF35-ORF36-HA containing a ΔG = −61 kcal/mol hairpin (Hp7) inserted after nucleotide position 32 in the native 5′ UTR. (D) 293T cells were transfected with the indicated WT or Hp7 plasmid shown in (C), and equivalent amounts of protein lysates were resolved by SDS-PAGE and Western blotted with anti-HA antibodies. S6RP served as a loading control. RNA samples were examined by Northern blot analysis with a ^32^P-labeled ORF36 DNA probe. GFP served as a co-transfection control. 18S rRNA was used as a loading control. (E) Schematic of 5′ UTR HA-ORF35-ORF36-HA indicating the nucleotide mutated to weaken the Kozak context flanking the ORF35 AUG (35 KCS wkn). (F) 293T cells were transfected with the indicated WT or 35 KCS wkn plasmid shown in (E), and equivalent amounts of protein lysates were resolved by SDS-PAGE and Western blotted with anti-HA antibodies. S6RP served as a loading control. RNA samples were examined by Northern blot analysis with a ^32^P-labeled ORF36 DNA probe. GFP served as a co-transfection control. 18S rRNA was used as a loading control.

The only other known example in KSHV of translation of a downstream ORF from a polycistronic mRNA occurs via an IRES [23]–[25]. We therefore used an established dual luciferase assay to determine whether an IRES similarly resides upstream of ORF36. The dual luciferase construct consists of a 5′-proximal *Renilla* luciferase gene that can be constitutively translated via a cap dependent mechanism, followed by a 3′-distal firefly luciferase gene, which is not normally translated. The two genes are separated by a defective encephalomyocarditis virus (ΔEMCV) to prevent translational read-through [11], [41]. Sequences of interest are then inserted between the ΔEMCV and the firefly luciferase gene, and IRES activity leads to the translation of firefly luciferase. Sequences encompassing ORF35, ORF35–36 or ORF34–36 as well as two known IRES elements (EMCV and KSHV ORF72) were cloned into the dual luciferase construct. The capped and polyadenylated *in vitro* transcribed mRNA was electroporated into lytically infected TREx BCBL1-RTA cells (Figure 3A). The integrity of the mRNAs was verified by Northern blotting (data not shown). After 4 h, the ratio of firefly/*Renilla* luciferase activity was measured to determine whether IRES activity was detectable in the context of lytic infection. Although both the EMCV and ORF72 control IRES elements supported translation of firefly luciferase, none of the sequences upstream of ORF36 possessed detectable IRES activity (Figure 3B).

We next sought to determine whether ORF36 translation was instead initiated via a cap-dependent mechanism by inserting a strong 40 nucleotide hairpin (Hp7; ΔG = −61 kcal/mol) after nucleotide 32 within the 72 nucleotide native 5′ UTR of the 5′ UTR HA-ORF35-ORF36-HA construct (Figure 3C) [42]. Stable hairpin structures (ΔG<−30 kcal/mol) present near the 5′ cap dramatically reduce translation initiation by stalling the pre-initiation complex [42]. Translation of both ORF35 and ORF36 was markedly reduced in the presence of Hp7 following either DNA or RNA transfection (Figure 3D, S1A). Thus, recognition of the 5′ cap and subsequent 40S scanning are critical for translation of both ORF35 and ORF36.

It is notable that ORF36 protein production is robust given that its translation requires the pre-initiation complex to bypass the relatively strong Kozak context surrounding the ORF35 start codon (AgaAUGG) and to scan through 424 nucleotides of upstream sequence. To determine whether the context of the ORF35 start codon influences the expression of ORF36, we mutated the preferred nucleotide (A) at position −3 to the least preferred nucleotide (U) (35 KCS wkn; Figure 3E). As expected, ORF35 expression was reduced; however, surprisingly, this mutation this did not significantly alter ORF36 expression, arguing against a pure leaky scanning mechanism to explain ribosomal access to the ORF36 start site (Figure 3F). Direct transfection with *in vitro* transcribed mRNAs confirmed that this result was not due to induction of an alternative promoter (Figure S1B). Thus, the relative strength of the ORF35 start site does not dramatically influence ORF36 translation, suggesting that there is an alternative mechanism in place that disfavors initiation at the 5′ gene.

### Two uORFs present in the 5′ UTR control translation of ORF35 and ORF36

**Figure 4:**
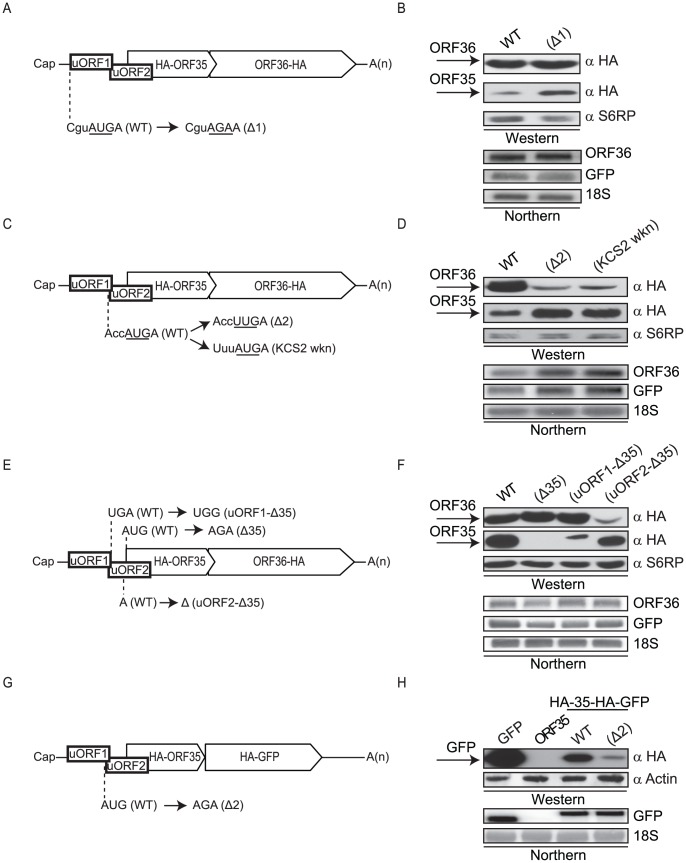
Two uORFs mediate translational control of ORF35 and ORF36. (A) Schematic representation of the uORF organization indicating the nucleotides mutated to disrupt the uORF1 AUG (Δ1). (B, D, F, H) 293T cells were transfected with the indicated wild-type or mutant plasmids, and 24 h post-transfection protein lysates were resolved by SDS-PAGE and Western blotted with anti-HA antibodies. S6RP or actin served as a loading control. RNA samples were examined by Northern blot analysis with a ^32^P-labeled ORF36 or GFP DNA probe. GFP served as a co-transfection control in B, D and F. 18S rRNA was used as a loading control. (C) Diagram indicating the nucleotide mutations used to disrupt (Δ2) or weaken (KCS2 wkn) the context of the uORF2 start codon. (E) The ORF35 start codon mutant (AUG→AGA; Δ35) and uORF fusion reporter RNAs are depicted schematically. uORF1-Δ35 has the uORF1 stop codon disrupted (UGA→UGG) while uORF2-Δ35 has one nucleotide deleted from uORF2 to shift the reading frame +1 (A→Δ). (G) Schematic of the bicistronic plasmid in which the ORF36 coding region was replaced with GFP. Because ORF36 partially overlaps with ORF35, this required truncating the C-terminus of ORF35. The uORF2 AUG mutation to AGA is also shown.

We searched for features of the ORF35–37 sequence that might contribute to translational start site selection. Within the 5′ UTR we noticed two short upstream ORFs (uORFs). The first nine codon uORF, dubbed uORF1, spans KSHV nucleotides 55603 to 55629 and has an AUG residing in a relatively weak Kozak context (CguAUGA) [18]. The second 11 codon uORF (uORF2) spans KSHV nucleotides 55626–55658 and overlaps with both the 3′ end of uORF1 and the ORF35 start codon (Figure 4A). To determine the contribution of uORF1 towards ORF35 and ORF36 translation, we mutated the uORF1 start site (Δ1) (Figure 4A). ORF35 expression was elevated in the Δ1 mutant (Figure 4B). We confirmed that the HA tag at the 5′ end of ORF35 did not alter this translational regulation by showing similar results upon repositioning of the HA tag internally within ORF35 (Figure S2). Thus, ORF35 expression undergoes modest negative regulation by ribosomal engagement at the uORF1 start codon, although this does not appear to influence ORF36 expression.

The uORF2 start codon is in a more favorable Kozak context than that of uORF1, and disruption of the uORF2 AUG (AUG→UUG; Δ2) or weakening the Kozak context of its start codon (KCS2 wkn) increased ORF35 translation and severely decreased translation of ORF36 in both DNA and RNA transfection experiments (Figure 4C–D, S3). Notably, the Δ2 mutant was designed to ensure the uORF1 stop codon remains intact, permitting the independent analysis of uORF1 and uORF2. Unlike uORF1, uORF2 therefore plays a key role in regulating expression of both genes in this polycistronic mRNA, likely due to the strong context flanking the uORF2 AUG as compared to the uORF1 start codon.

Although a few rare uORFs have been found to function in a sequence-dependent manner [43]–[47], for most characterized uORFs it is the act of translation rather than the peptide sequence that mediates their function. The fact that 45% of the uORF2 amino acid sequence is altered in the construct bearing the HA tag at the 5′ end of ORF35 is in agreement with the amino acid sequence of uORF2 not being the primary determinant of its activity. Indeed, rebuilding the uORF2 mutants into a construct in which the HA tag was moved to an internal position in ORF35 yielded indistinguishable results (Figure S4).

The above findings suggested that engagement of the translation machinery at either uORF1 or uORF2 rather than the sequence of the uORF-encoded peptide mediates their regulatory function. We therefore sought to confirm that these uORFs were indeed recognized by the translation machinery. Due to their small size, uORF-generated peptides tend to be highly unstable and are very difficult to detect. To circumvent this problem, we made a single nucleotide change in each uORF to place them in frame with ORF35 lacking its AUG (Δ35), thereby generating uORF-ORF35 fusions (Figure 4E). Thus, restoration of ORF35 expression is a direct readout translation initiation from the uORF start codon. In both cases, the uORF fusions restored ORF35 expression to levels corresponding to the relative strength of the Kozak consensus sequence of each uORF (Figure 4F). As expected, only the uORF2 fusion abrogated expression of ORF36 (Figure 4F).

Finally, to determine whether additional *cis*-acting elements within ORF36 are required for its translation after uORF2 engagement, we replaced the ORF36 gene with a GFP reporter (Figure 4G). GFP protein was expressed robustly as a downstream gene from this construct, arguing against a requirement for an element within ORF36 for its translation (Figure 4H). Similar to our results with ORF36, disruption of uORF2 compromised expression of GFP (Figure 4H), supporting a uORF2-dependent mechanism as the primary pathway enabling translation of a downstream gene from this locus.

### ORF36 expression occurs via reinitiation after uORF translation

**Figure 5:**
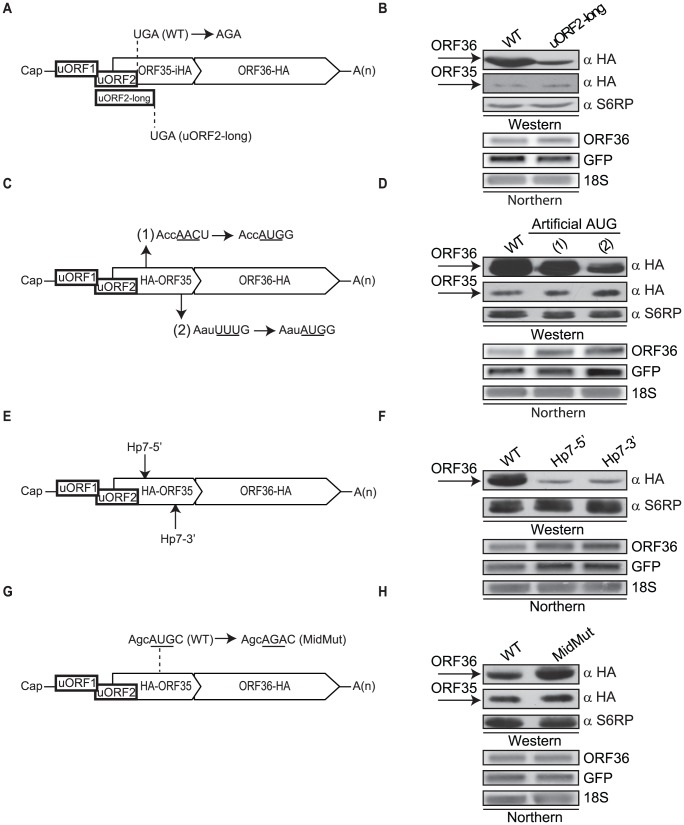
Ribosomal access to the ORF36 start codon occurs via linear scanning after termination of uORF translation. (A) Schematic of the elongation of uORF2. The uORF2 stop codon and the four subsequent in-frame stop codons were mutated, artificially lengthening uORF2 from 11 to 64 amino acids. (B, D, F, H) 293T cells were transfected with the indicated wild-type or mutant plasmids, and 24 h post-transfection protein lysates were resolved by SDS-PAGE and Western blotted with anti-HA antibodies. S6RP served as a loading control. RNA samples were examined by Northern blot analysis with a ^32^P-labeled ORF36 DNA probe. GFP served as a co-transfection control. 18S rRNA was used as a loading control. (C) Schematic of AUG insertions at two locations in the ORF35 coding region, placed out of frame with ORF36. All AUGs were designed to have the two dominant Kozak consensus sequence nucleotides (A at −3 and G at +4). (E) Schematic of the wild-type 5′ UTR-HA-ORF35-ORF36-HA construct showing the location of the Hp7 insertion into the 5′ or 3′-proximal region of the ORF35 coding region. (G) Schematic of the wild-type 5′ UTR-HA-ORF35-ORF36-HA construct showing the location of the native AUG within the ORF35 codon region which has been mutated to AGA to generate the MidMut construct.

Translation of a major ORF following engagement at a uORF generally occurs via a termination-reinitiation event. The length of a uORF is important for reinitiation, as it is thought that some of the translation initiation accessory factors have not yet dissociated prior to termination at the uORF stop codon [4]. In this regard, translation of the downstream ORF decreases dramatically if the time required to complete translation of the uORF is increased, for example by increasing the ORF length or inserting secondary structure to stall the ribosome [48], [49]. Therefore, we reasoned that if ORF36 translation initiates using the same 40S ribosomal subunit involved in translation of uORF2, then artificially elongating uORF2 should inhibit ORF36 expression. This experiment was performed on the construct backbone with the ORF35 HA tag located internally to mimic the wild type length of uORF2. Indeed, extension of uORF2 from 11 to 64 codons (uORF2-long) resulted in a dramatic drop in ORF36 expression (Figure 5A–B).

The rate-limiting step of reinitiation is postulated to be the re-acquisition of the pre-initiation complex (eIF2-GTP-Met-tRNA_i_) during ribosomal scanning, and thus a sequence of sufficient length must be present downstream of the uORF for this to occur [3], [4]. We therefore evaluated how the distance between the uORF2 stop codon and the subsequent start codon influences reinitiation within the viral mRNA. Start codons in a favorable Kozak context were inserted at two positions between the uORF2 stop codon and the ORF36 start site. We hypothesized that start codons located close to uORF2 would not be as efficiently recognized, and therefore they would not inhibit ORF36 expression. However, more distally located start codons should better engage the initiation machinery, thereby preventing translation from occurring at the authentic ORF36 start site. In agreement with this prediction, a start codon positioned 16 nucleotides downstream of uORF2 did not strongly inhibit ORF36 expression, whereas a methionine positioned 246 nucleotides after termination of uORF2 severely compromised ORF36 expression (Figure 5C–D). These data support the conclusion that engagement of the ORF36 start codon is dependent on the reacquisition of the pre-initiation complex after termination of uORF2 translation.

### The ORF36 start codon is accessed by linear scanning

Translation reinitiation at the internal ORF36 start codon could occur either after linear scanning of the 40S complex through the 332-nucleotide intercistronic region between uORF2 and ORF36 or through shunting of the complex past this sequence and its subsequent positioning proximal to ORF36. To distinguish between these possibilities, two strong hairpins (Hp7) that impede scanning were inserted within the 5′-proximal or 3′-proximal coding region of ORF35 (Figure 5E). If the 40S ribosomal subunit were shunted past these internal sequences, one or both of the hairpins (depending on the location of the shunting sites) should not compromise ORF36 translation [5], [50]. However, we observed a significant reduction in ORF36 expression in the presence of either hairpin, arguing that the 40S complex scans in a linear fashion through ORF35 (Figure 5F).

One potential caveat is that the insertion of the hairpins might dramatically alter the RNA folding landscape, disrupting a secondary structure required for shunting. To exclude this possibility, the single natural methionine codon present within the coding region of ORF35, was mutated to an arginine (MidMut; Figure 5G). If this internal sequence were bypassed via shunting after uORF2 termination, the natural start codon should not be able to compete with the ORF36 AUG for the pre-initiation complex. However, we found that ORF36 expression was increased from the MidMut construct, arguing against a shunting mechanism and further suggesting that this methionine normally engages a fraction of the scanning ribosomes before they can reach the ORF36 start codon (Figure 5H). Translation of the peptide generated cannot be directly monitored due to the fact that it is only eight amino acids. Collectively, these data support a model in which the preferential recognition of uORF2 diverts ribosomes past the ORF35 start codon, whereupon they scan in a linear fashion and reacquire the pre-initiation complex before reinitiating translation at a downstream start codon.

### Disruption of uORF2 alters ORF36 expression during lytic infection

**Figure 6:**
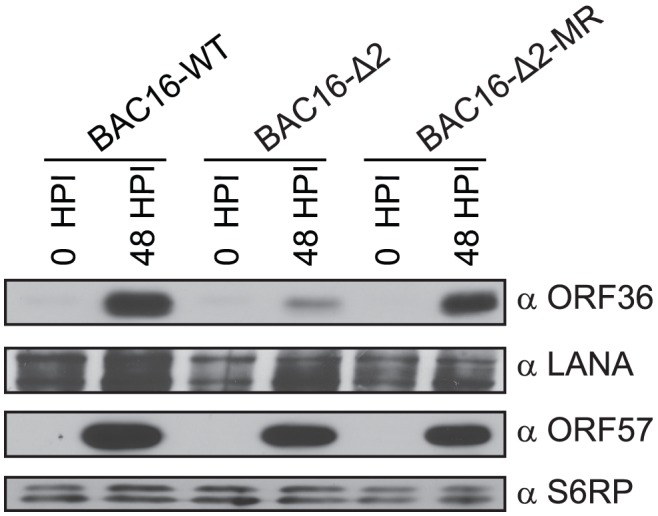
Disruption of uORF2 alters ORF36 expression during lytic infection. iSLK-PURO cells stably harboring the WT KSHV BAC16, a uORF2 mutant BAC16 (BAC16-Δ2), or a mutant rescue BAC16 (BAC16-Δ2-MR) were either untreated or lytically reactivated for 48 h. Protein lysates were Western blotted with antibodies against ORF36, the viral latent protein LANA and a viral lytic protein ORF57. S6RP served as a loading control.

To confirm that uORF2 regulates ORF36 expression during lytic KSHV infection, we engineered a uORF2 point mutant (BAC16-Δ2; ATG→TTG) and a revertant mutant rescue (BAC16-Δ2-MR; TTG→ATG) within the recently described KSHV BAC16 (Figure S5) [51]. BAC16-WT, BAC16-Δ2 and BAC16-Δ2-MR were transfected into iSLK-PURO cells bearing a doxycycline-inducible RTA expression system to enable lytic reactivation [52]. Immunoblot analysis using polyclonal anti-sera specific for ORF36 revealed that while ORF36 was readily detectable at 48 h post-lytic reactivation in cells infected with WT or the mutant rescue virus, deletion of the uORF2 start codon severely compromised ORF36 expression (Figure 6). In contrast, the uORF2 mutation had no effect on the levels of the KSHV latent protein LANA or the lytic protein ORF57, confirming its specificity for ORF36 (Figure 6). Thus, uORF2 plays a critical role in enabling expression of the ORF36-encoded viral protein kinase during lytic KSHV infection.

### Conservation of uORFs within related γ-herpesviruses

**Figure 7:**
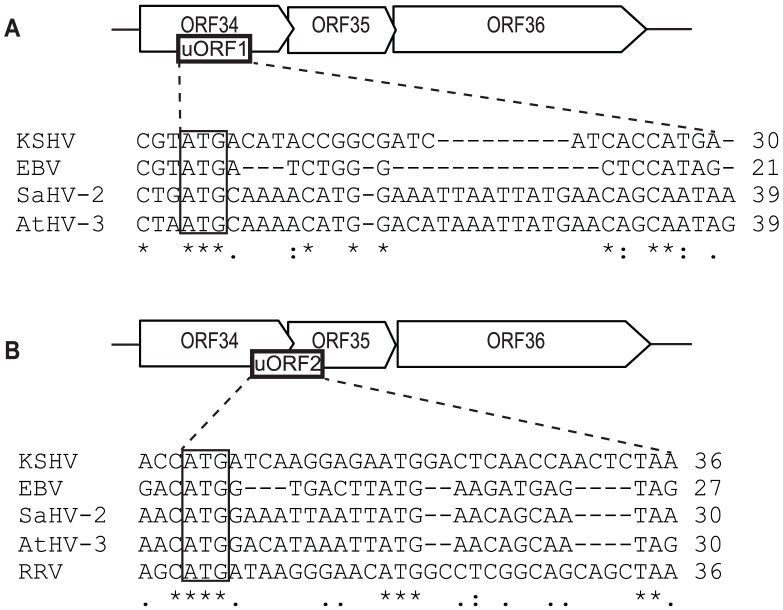
uORF1 and uORF2 are conserved among select γ-herpesviruses. Alignment using ClustalW2 of (A) uORF1 or (B) uORF2 from KSHV, EBV, SaHV-2, AtHV-3 and RRV. Consensus nucleotides are indicated (three: asterisk; two: dot). uORF length is indicated on the right, and the uORF start codons are boxed.

We examined whether the loci analogous to KSHV ORF35–37 in several additional γ-herpesviruses also possessed uORFs within their 5′ UTRs (Table S1). Indeed, we identified two 6–12 codon uORFs within the predicted 5′ UTR of the locus in Epstein Barr virus (EBV), herpesvirus saimiri (HaSV-2) and ateline herpesvirus 3 (AtHV-3) and one 11 codon uORF in good context within the 5′ UTR of the rhesus rhadinovirus (RRV) locus (Figure 7A, 7B). The fact that the uORF positioning but not the coding sequence is conserved supports the hypothesis that their regulatory contribution relies on their ability to engage translation complexes, rather than the actual peptide produced. Furthermore, eight of the nine ORF35 homologs examined contain ≤2 internal methionine codons, as would be predicted if a termination-reinitiation mechanism was used to translate the downstream gene (Table S1). Interestingly, in all cases where two uORFs are present, the first uORF is within a weaker Kozak context than the second uORF, which overlaps the start codon of each ORF35 homolog (EBV BGLF3.5, SaHV-2 ORF35, AtHV-3 ORF35 and RRV ORF35). Thus, the conservation of uORFs at this genetic locus suggests that using uORFs to enable expression of a 3′-proximal gene may be a conserved strategy for translational control among these viruses. However, whether these loci indeed encode a functional polycistronic mRNA and are regulated by a similar uORF-based mechanism remains to be experimentally verified.

## Discussion

**Figure 8:**
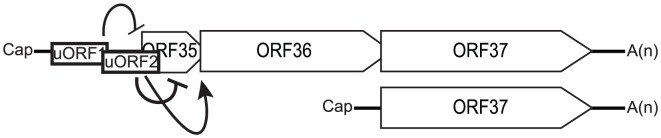
Model of the mechanisms of translation initiation used to translate ORF35, ORF36 and ORF37. ORF35 translation is repressed by uORF1 and uORF2. ORF36 is translated from a termination-reinitiation event after translation of uORF2. ORF37 is translated from an ORF37-specific transcript generated from a promoter located within the coding region of ORF36.

In this study, we describe a novel functionally bicistronic viral mRNA that is translated via a unique adaption of ribosomal reinitiation. In other characterized examples of viral translation via a reinitiation mechanism, expression of the downstream gene is significantly tempered as a consequence of ribosomal engagement at an upstream start codon [43], [53]–[56]. Aside from being bicistronic, translation from the KSHV ORF35–37 transcript is unusual in that the protein product of ORF36 is at least as robustly expressed as the 5′ ORF35 despite the fact that the ORF35 start codon is in a favorable sequence context. We reveal that a key mechanism underlying this phenotype involves the position of a short uORF overlapping the start codon of ORF35, which enables translation of ORF36 (Figure 8). These findings provide the first example of cap-dependent non-canonical translation in KSHV and illustrate a novel strategy to translate polycistronic mRNA.

Several lines of evidence support the notion that ORF36 is expressed in a cap-dependent manner as a 3′-proximal cistron. No transcript of an appropriate size with ORF36 as the 5′-proximal cistron was detected in KSHV-infected cells, in agreement with the results of 5′ RACE that indicated its transcription starts upstream of ORF35 [26]. In addition, ORF36 protein expression was detected after transfection of an *in vitro* transcribed bicistronic RNA transcript. Finally, interfering with scanning from the 5′ mRNA cap via insertion of a hairpin blocked ORF36 translation, consistent with our failure to detect IRES activity within the locus. This is in contrast with the sole functionally bicistronic KSHV mRNA described to date, where an IRES is present within the coding region of ORF72 allows for ORF71 expression in a cap-independent manner [23]–[25].

Our results indicate that the ORF36 start codon is accessed via a termination-reinitiation event after translation of uORF2. The most 5′ uORF (uORF1) resides in a weaker context than uORF2, which overlaps the ORF35 start codon. Importantly, because the stop codon of uORF1 overlaps with the start site of uORF2, engagement of these uORFs is mutually exclusive. Therefore, preferential initiation at uORF2 likely drives the enhanced translation of ORF36 by causing ribosomes to bypass the favorable ORF35 start codon. After translating uORF2, ribosomes continue to scan through the following 332 nucleotides to reinitiate at ORF36. In support of this model, lengthening uORF2 to decrease the efficiency of reinitiation abrogated ORF36 expression. Furthermore, weakening the context surrounding the uORF2 start codon enhanced ORF35 expression, suggesting that the ORF35 start site is primarily reached by ribosomes that have bypassed the AUG of uORF2, likely by leaky scanning. This provides a rare example of a uORF enhancing translation of a downstream major ORF.

To date, the only described short uORF that enables access to the start codon of a downstream gene in a polycistronic transcript was identified in hepatitis B virus (HBV). The HBV uORF, dubbed C0, weakly inhibits the 5′-proximal C ORF while stimulating translation of the 3′-proximal J and P proteins [6], [57]. However, the termination-reinitiation event described for HBV may be facilitated by a shunting mechanism, as non-linear scanning was found to occur in the homologous region in the related duck hepatitis B virus [58]. This appears not to be the case for ORF36 because insertion of strong hairpins within the coding region upstream strongly compromises ORF36 expression, suggesting that the ribosomes are scanning continuously from the 5′ mRNA cap to the ORF36 start codon.

uORFs are common features found in the 5′ UTRs of many mammalian mRNAs [59]. They are widely recognized as *cis*-regulatory elements and their presence generally correlates with reduced translation of the major ORF by causing initiation to instead occur by leaky scanning or a low-efficiency reinitiation event, which is agreement with the function of uORF1 as a negative regulator of ORF35 [4], [59], [60]. A few cases have been described in which the ability of the uORF to repress downstream translation is dependent on the amino acid sequence of the encoded peptide [43]–[47]. For example, a uORF present in the 5′ UTR of the human cytomegalovirus gp48 gene attenuates downstream translation in a sequence-dependent fashion, likely by delaying normal termination and preventing leaky scanning by the 40S ribosomal subunit to reach the downstream AUG [43]. However, in general, engagement of the translation apparatus rather than the translated product itself represses translation of the major ORF. Indeed, regulation of the ORF35–37 transcript appears independent of the uORF peptide sequence because the 5′ HA-tagged construct had two amino acids mutated within uORF2 yet still functioned to permit translation of ORF36. Moreover, uORFs in homologous regions of the genome in related γ-herpesviruses lacked amino acid conservation. However, individual amino acid substitutions in all of the uORF1 and uORF2 codons would be required to formally rule out a role for the encoded peptides in the translational control of this mRNA.

Factors that influence the ability of a terminating ribosome to resume scanning remain poorly understood. It has been shown using chimeric preproinsulin mRNAs that efficient reinitiation progressively improves upon lengthening the intercistronic sequence up to 79 nucleotides [61]. Sufficient intercistronic sequence length is thought to be necessary to allow time for the scanning 40S ribosomal subunit to reacquire eIF2-GTP-Met-tRNA_i_ prior to encountering the downstream start codon, although at what point the sequence length becomes inhibitory is not known [4], [49]. In the context of the viral ORF35–37 transcript, the ribosome is able to reinitiate translation with a high frequency despite scanning 332 nucleotides after terminating translation of uORF2, indicating that intergenic regions significantly longer than 79 nucleotides still enable reinitiation.

Interestingly, a prior report identified a translational enhancer element within the tricistronic S1 mRNA of avian reovirus that functions to increase expression of a downstream cistron. This occurs as a consequence of sequence complementarity to 18S rRNA, which is reminiscent of the prokaryotic Shine-Dalgarno sequence [62], [63]. A similar strategy of having 18S rRNA complementarity within a bicistronic mRNA was also found to enhance the ability of the minor calicivirus capsid protein VP2 to be translated by reinitiation [56], [64]. Whether enhancer elements exist in the KSHV uORF-ORF36 intercistronic region to facilitate translation at the downstream cistron remains to be determined. However, no critical reinitiation element exists downstream of the ORF36 start codon, as replacement of these sequences with GFP does not block its translation. This is distinct from the termination-reinitiation mechanism described for certain retrotransposons, which require complex downstream secondary structures [65].

The question arises as to what benefit is conferred by this finely tuned strategy of translational control for both ORF35 and ORF36. One possibility is that ORF35 and ORF36 are required at different points during lytic infection and that during the course of viral replication, conditions arise that favor translation of one protein versus the other. This type of regulation occurs in the well-characterized *Saccharomyces cerevisiae* GCN4 locus, where four short uORFs modulate reinitiation at the major ORF depending on the level of eIF2α phosphorylation [66]–[68]. Indeed, certain types of cell stress have also been shown to influence non-canonical translation of the cytomegalovirus UL138 gene [69]. Alternatively, the uORFs may confer a tight level of regulation to ensure that ORF36 is not synthesized at deleterious levels during infection. For example, an EBV mutant that over-produces BGLF4 (the ORF36 homolog) exhibited defects in viral replication [39]. Determining if and how this non-canonical mechanism of translational control influences the KSHV lifecycle will be an important future endeavor.

## Materials and Methods

### Plasmid constructs

pcDNA3.1(+)-ORF35–37 was generated by PCR-amplifying the ORF35–37 genetic locus from the KSHV-BAC36 (kindly provided by G. Pari [70]) and cloning it into the EcoRI/NotI sites of pcDNA3.1(+) (Invitrogen). pcDNA3.1(+)-5′ UTR-HA-ORF35 was assembled in a two-step process starting with the addition of the N-terminal HA tag after the native start ATG (nucleotide sequence: GCTTACCCATACGATGTAC CTGACTATGCG) to the coding sequence amplified from the KSHV genome as above, followed by an overlap extension PCR to insert the 72 nucleotide (nt) native 5′ UTR. The final product was then inserted into the pcDNA3.1(+) EcoRI/NotI restriction sites. pcDNA3.1(+)-ORF36 was constructed by PCR-amplification of the ORF36 coding sequence or to add the in frame C-terminal HA tag (GCTTACCCATACGATGTACCTG ACTATGCGTGA) followed by insertion into EcoR1/Not1 restriction sites. pCDEF3-ORF37 is described elsewhere [37]. HA-ORF35-ORF36-HA was amplified from the KSHV-BAC36 using primers with additional HA tag sequences and inserted into the EcoR1/Not1 sites of pcDNA3.1(+). This was followed by scarless insertion of the native 5′ UTR via two-step sequential overlap extension PCR [70]. To construct 5′ UTR-ORF35iHA-ORF36-HA, a backbone construct consisting of 5′UTR ORF35-ORF36-HA was first generated by PCR-amplification from the KSHV-BAC36 with HA tag sequences solely for ORF36 and inserted into the EcoR1/Not1 sites of pcDNA3.1(+). This construct was then linearized by inverse PCR at nucleotide position 55795 followed by ligation-independent cloning using InFusion (Clonetech) with primers consisting of an HA tag flanked by 15 base pair regions of vector overlap. A stable hairpin structure (Hp7 sequence: GGGGCGCGTGGTGGCGGCTGCAGCCGCCACCACGCGCCCC, [42]) was inserted into the 5′ UTR at nucleotide position 55599, or within the ORF35 coding region at nucleotide position 55662 and at position 55862 [18]. For the 5′ UTR HA-ORF35Δ96-HA-GFP construct, HA-GFP was inserted between the NotI/XbaI restriction sites in pcDNA3.1(+), and the 5′ UTR-HA-ORF35 Δ96 fragment was then inserted between the EcoRI/NotI restriction sites upstream of HA-GFP. Two bicistronic, dual luciferase constructs, a negative control (ΔEMCV; mutated IRES sequence) and a positive control (ΔEMCV element+functional EMCV) were kindly provided by P. Sarnow (Stanford University) [11], [41]. ORF72, ORF34–36, ORF35–36 and ORF35 PCR amplicons were inserted into the EcoRI restriction site downstream of the ΔEMCV element and upstream of firefly luciferase. The primers used to generate these constructs are listed in Table S2.

Where specified, parental plasmids were subjected to site-directed mutagenesis using the QuikChange kit (Stratagene) as per the manufacturer's protocol. The context of the ORF35 start codon was weakened by mutating the wild type AgaAUGG to UgaAUGG (35 KCS wkn). uORF1 and uORF2 mutants (designated Δ1 and Δ2) were generated by substituting the AUG start codon with AGA or UGA, respectively. The uORF2 Kozak context was weakened by mutating the wild-type AccAUGA to UuuAUGA (KCS2 wkn). The ORF35 start codon was disrupted by mutating the wild type AUG to AGA (Δ35). The uORF1 fusion to Δ35 was generated by mutating the uORF1 stop codon UGA to UGG (uORF1-Δ35). The uORF2 fusion to Δ35 was generated by deleting one nucleotide (A) located immediately prior to the ORF35 start codon (uORF2-Δ35). Two codons within in the ORF35 coding region were converted to AUGs in a strong context: (1) AccAACU to AccAUGG and (2) AauUUUG to AauAUGG. The native AUG residing at location 55778-80 within the ORF35 coding region was mutated to an AGA (MidMut) [18]. uORF2 was lengthened from 11 to 64 codons by mutating the first UAA stop codon to AGA, the second UAA stop codon to CAA, the third UGA stop codon to CGA, and the fourth and fifth UAG stop codon to CAG, resulting in the use of the next downstream stop codon (uORF2-long).

### BAC mutagenesis and DNA isolation

The KSHV BAC16 was modified as described previously [51] use a two-step scarless Red recombination system [71]. Briefly, BAC16 was introduced in GS1783 *E. coli* strain by electroporation (0.1 cm cuvette, 1.8 kV, 200 Ω 25 µF). A linear DNA fragment encompassing a kanamycin resistance expression cassette, an I-SceI restriction site and flanking sequence derived from KSHV genomic DNA was generated by PCR and subsequently electroporated into GS1783 *E. coli* harboring BAC16 and transiently expressing *gam*, *bet* and *exo*. Integration of the Kan^R^/I-SceI cassette was verified by PCR and restriction enzyme digestion of the purified BAC16 DNA. The second recombination event between the duplicated sequences resulted in the loss Kan^R^/I-SceI cassette and the seamless recirculation of the BAC16 DNA, yielding kanamycin-sensitive colonies that were screened by replica plating. BAC16 DNA was purified from chloramphenicol-resistant colonies using the NucleoBond 100 (Machery-Nagel) as per the manufactures instructions.

### Cells, transfections and drug treatment

Human embryonic kidney 293T cells were maintained in Dulbecco's modified Eagle's medium (DMEM) supplemented with 10% fetal bovine serum (FBS) (Gibco). The iSLK-PURO KSHV-negative endothelial cell lines [51], [52] were maintained in DMEM supplemented with 10% FBS, penicillin (100 U/ml, Gibco) and streptomycin (100 µg/ml, Gibco). To induce lytic reactivation of KSHV, iSLK-PURO cells were treated with doxycycline (1 µg/ml, BD Biosciences) and sodium butyrate (1 mM, Sigma). TREx BCBL1-RTA [72] cells were maintained in RPMI supplemented with 10% FBS, L-glutamine (200 µM, Invitrogen), penicillin (100 U/ml), streptomycin (100 µg/ml) and hygromycin B (50 µg/ml, Omega Scientific). To induce lytic reactivation of KSHV, TREx BCBL1-RTA cells were split to 1×10^6^ cells/ml and induced 24 h later with 2-O-tetradecanoylphorbol-13-acetate (TPA; 20 ng/ml, Sigma), doxycycline (1 µg/ml) and ionomycin (500 ng/ml, Fisher Scientific) [73].

For DNA transfections, constructs (1 µg/ml) were transfected into subconfluent 293T cells grown in 12-well plates, either alone or in combination with 0.1 µg/ml GFP as a co-transfection control using Effectene reagent (Qiagen) or Lipofectamine 2000 (Invitrogen) following the manufacturers protocols. For RNA transfections, 3 µg/ml of mRNA *in vitro* transcribed using the mMessage mMachine kit (Ambion) and polyadenylated with yeast poly(A) polymerase (Epicentre Technologies) was transfected into ∼90% confluent 293T cells grown in 12-well plates using Lipofectamine 2000. TREx BCBL1-RTA cells were transfected with 20 µg of DNA per 10^7^ cells via electroporation (250 V, 960 µF) with a Gene Pulser II (Bio-Rad, Hercules, CA).

For BAC transfections and reconstitution, ∼70% confluent iSLK-PURO cells were grown in a 24-well plate followed by transfection with 500 ng of BAC DNA via FuGENE 6 (Promega), after 6 h, a further 500 ng BAC DNA was transfected with Effectene, following the manufacturers protocols and subsequently selected with 800 µg/ml hygromycin B to establish a pure population. iSLK-PURO-BAC16 cells were then induced with doxycycline (1 µg/mL) and sodium butyrate (1 mM) to enter the lytic cycle of KSHV replication.

### Luciferase assays

Luciferase activities were determined using the dual-luciferase assay system (Promega) and a bench-top luminometer according to manufacturer's protocol. IRES activity was calculated by obtaining the firefly/*Renilla* activity ratios for each of constructs containing the putative IRES sequences or the positive controls and dividing them by the ratio obtained from the ΔEMCV negative control. The value of fold activation represents at least three independent experiments with triplicate samples in each electroporation. Error bars represent the standard deviation between replicates.

### Western and Northern blots

Protein lysates were prepared in RIPA buffer [50 mM Tris-HCl (pH 8.0), 150 mM NaCl, 1% (v/v) Nonidet P-40, 0.5% (w/v) sodium deoxycholate, 0.1% (w/v) sodium dodecyl sulfate (SDS)] containing protease inhibitors (Roche), and quantified by Bradford assay. Equivalent quantities of each sample were resolved by SDS-PAGE, transferred to a polyvinylidene difluoride membrane and incubated with the following primary antibodies: mouse monoclonal GFP (1∶2000, BD Biosciences), mouse monoclonal HA (1∶2000, Invitrogen), rabbit polyclonal ORF36 (1∶5000, kindly provided by Y. Izumiya [27]), goat polyclonal horseradish peroxidase (HRP)-conjugated actin (1∶500, Santa Cruz Biotechnology), rabbit polyclonal SOX J5803 (1∶5000, [38]), rabbit polyclonal ORF57 (1∶5000, kindly provided by Z. Zheng [74], rabbit polyclonal LANA #6 (1∶1000) or mouse monoclonal S6RP (1∶1000, Cell Signaling) followed by incubation with HRP-conjugated goat anti-mouse or goat anti-rabbit secondary antibodies (1∶5000 dilution) (Southern Biotechnology Associates).

Total cellular RNA was isolated for Northern blotting using RNA-Bee (Tel-Test). The RNA was then resolved on 1.2–1.5% agarose-formaldehyde gels, transferred to Nytran nylon membranes (Whatman) and probed with ^32^P-labeled DNA probes made using either the RediPrime II random prime labeling kit (GE Healthcare) or the Decaprime II kit (Ambion). Strand-specific riboprobes specific for ORF36 and ORF37 were synthesized using the Maxiscript T7 kit (Ambion) with ^32^P-labelled UTP. The probes used for Northern blot analysis spanned the following regions according to the nucleotide positions described by Russo et al. [18]: ORF35 probe: 55639–56091, ORF36 full-length probe: 55976–57310: ORF36-specific probe: 56093–56805: and ORF37 probe: 57273–58733. Results in each figure are representative of at least three independent replicates of each experiment.

### Sequence alignments

The uORF1 and uORF2 alignments were generated from data obtained from the NIAID Virus Pathogen Database and Analysis Resource (ViPR) online through the web site at http://www.viprbrc.org.

## Supporting Information

Figure S1 **Translation of ORF36 is dependent on the 5′ mRNA cap yet not strongly inhibited by the ORF35 start codon.** (A–B) 293T cells were transfected with the indicated *in vitro* transcribed capped and polyadenylated RNA. The wild type construct consists of 5′UTR HA-ORF35-ORF36-HA. Hp7 contains a ΔG = −61 kcal/mol hairpin inserted after nucleotide position 32 in the native 5′ UTR. 35 KCS wkn was generated by mutating AgaAUGG→UgaAUGG to weaken the Kozak context flanking the ORF35 AUG. Protein lysates were harvested 4 h post-transfection, resolved by SDS-PAGE and detected with anti-HA antibodies. The ribosomal protein S6RP served as a loading control for both experiments.
(EPS)

Figure S2 **The location of the HA tag does not influence ORF35 expression or uORF1 regulation of ORF35.** (A) Schematic representation of the uORF1 mutations introduced into a construct with the native 5′ UTR-ORF35-ORF36-HA with an HA tag positioned internally and in-frame with ORF35 (WT-iHA). (B) 293T cells were co-transfected with the indicated WT-iHA, Δ1-iHA and GFP. Protein lysates were harvested 24 h post transfection, resolved by SDS-PAGE and Western blotted with anti-HA antibodies to detect both ORF35 and ORF36. S6RP served as a loading control. RNA samples were examined by Northern blot analysis with a ^32^P-labeled ORF36 DNA probe. GFP served as a co-transfection control. 18S rRNA was used as a loading control.
(EPS)

Figure S3 **uORF2 regulates translation of ORF35 and ORF36.** (A) Diagram indicating the nucleotide mutations used to disrupt (Δ2) or weaken (KCS2 wkn) the context of the uORF2 start codon. (B) 293T cells were transfected with *in vitro* transcribed capped and polyadenylated RNA to compare the wild type bicistronic mRNA with the uORF2 start codon mutants. Protein lysates were harvested 4 h post-transfection, resolved by SDS-PAGE and detected with anti-HA antibodies. The ribosomal protein S6RP served as a loading control for both experiments.
(EPS)

Figure S4 **The location of the HA tag does not influence bicistronic coding capacity.** (A) Schematic representation of the uORF2 mutations introduced into a construct with the native 5′ UTR-ORF35-ORF36-HA with an HA tag positioned internally and in-frame with ORF35 (WT-iHA). (B) 293T cells were co-transfected with the indicated WT-iHA, Δ2-iHA or KCS2 wkn-iHA and GFP. Protein lysates were harvested 24 h post transfection, resolved by SDS-PAGE and Western blotted with anti-HA antibodies to detect both ORF35 and ORF36. S6RP served as a loading control. RNA samples were examined by Northern blot analysis with a ^32^P-labeled ORF36 DNA probe. GFP served as a co-transfection control. 18S rRNA was used as a loading control.
(EPS)

Figure S5 **Analysis of BAC16 uORF2 mutant and mutant rescue clones.** BAC16 WT, uORF2 mutant (BAC16-Δ2), or mutant rescue (BAC16-Δ2-MR) DNA was isolated from GS1783 *Escherichia coli*, digested with *NheI* and subjected to pulse-field gel electrophoresis. M, 1 Kb marker (Biorad) and MidRange I PFG marker (NEB). Expected fragment sizes in base pairs: 35000, 28862, 25693, 20742, 9062, 8852, 7788, 7575, 6376, 5879, 5011, 4739, 4553, 4378, 3838 and 1663. *NheI* digestion does not introduce or alter any *NheI* recognition sites.
(EPS)

Table S1 **Analysis of the region upstream of ORF35 in the genomes of γ-herpesviruses with the conserved** ***ORF34–37*** **genetic locus.** A representative strain of each γ-herpesvirus deposited in the Virus Pathogen Database and Analysis Resource that retains the arrangement of *ORF34–37* genetic locus was included in the sequence analysis. The region upstream of the ORF35 start codon (≤100 nucleotides) was used as an arbitrary prediction of the 5′UTR. The number of internal AUG codons represents those located between the uORF2 stop codon and the start codon of ORF36 within each respective mRNA.
(DOCX)

Table S2 **List of oligonucleotide primers.** List of primer used to generate constructs in this study.
(DOCX)
