# Supplementary material for: Dual Short Upstream Open Reading Frames Control Translation of a Herpesviral Polycistronic mRNA
Source: PLoS Pathog. 2013 Jan 31;9(1):e1003156. doi: 10.1371/journal.ppat.1003156 (PMC3561293; doi:10.1371/journal.ppat.1003156)
Supplement: Table S1 — Analysis of the region upstream of ORF35 in the genomes of γ-herpesviruses with the conserved ORF34–37 genetic locus. A representative strain of each γ-herpesvirus deposited in the Virus Pathogen Database and Analysis Resource that retains the arrangement of ORF34–37 genetic locus was included in the sequence analysis. The region upstream of the ORF35 start codon (≤100 nucleotides) was used as an arbitrary prediction of the 5′UTR. The number of internal AUG codons represents those located between the uORF2 stop codon and the start codon of ORF36 within each respective mRNA. (DOCX) [file ppat.1003156.s006.docx]

**Table S1. Analysis of the region upstream of ORF35 in the genomes of**

**γ-herpesviruses with the conserved *ORF34-37* genetic locus.**

| **Name** | **Strain** | **GenBank Accession #** | **uORF1** | **uORF2** | **# of internal AUG codons** |
| --- | --- | --- | --- | --- | --- |
| HHV-8 (KSHV) | GK18(P) | NC_009333 | Yes, weak* | Yes, strong* | 2 |
| HHV-4 (EBV) | AG876 | NC_009334 | Yes, weak* | Yes, strong* | 0 |
| Saimiriine herpesvirus 2 (SaHV-2) | C488 | AJ410493 | Yes, weak* | Yes, strong* | 2 |
| Ateline herpesvirus 3 (AtHV-3) | 73 | NC_001987 | Yes, weak* | Yes, strong* | 2 |
| Macacine herpesvirus 5 (RRV) | 17577 | NC_003401 | No | Yes, strong* | 1 |
| Murid herpesvirus 4 | WM8 | GQ169129 | No | Yes, weak* | 4 |
| Equid herpesvirus 2 | 86/87 | NC_001650 | No | No | 0 |
| Ovine herpesvirus 2 | UNKNOWN | DQ198083 | No | No | 0 |
| Alcelaphine herpesvirus 1 | C500 | AF005370 | No | No | 1 |

*weak: Lacks a purine at the -3 position of the Kozak consensus sequence.

*strong: Presence of a purine at the -3 position of the Kozak consensus sequence.
