# Supplementary material for: Dual Short Upstream Open Reading Frames Control Translation of a Herpesviral Polycistronic mRNA
Source: PLoS Pathog. 2013 Jan 31;9(1):e1003156. doi: 10.1371/journal.ppat.1003156 (PMC3561293; doi:10.1371/journal.ppat.1003156)
Supplement: Table S2 — List of oligonucleotide primers. List of primer used to generate constructs in this study. (DOCX) [file ppat.1003156.s007.docx]

**Table S2. List of oligonucleotide primers.**

| **Primer Name** | **Primer Sequence 5’-3’** |
| --- | --- |
| **ORF35-37 F** | GCGAATTCGTCCAACTACGGGCGACTATCT |
| **ORF35-37 R** | GAGCTCGCGGCCGCAATGCGGGATATGGGGATTGC |
| **HA-ORF35 F** | GGGCCCGAATTCATGGCTTACCCATACGATGTACCTGACTATGCGGACTCAACCAACTCTAAAAGAG |
| **ORF35 R** | GAGCTCGCGGCCGCTTAGGGAGTTTCAGGGCACAC |
| **ORF36 F** | GGGCCCGAATTCATGGAGAGGAGACCCCCACTC |
| **ORF36 R** | GAGCTCGCGGCCGCTCAGAAAACAAGTCCGCGGGTG |
| **ORF36-HA R** | GAGCTCGCGGCCGCTCACGCATAGTCAGGTACATCGTATGGG |
| **ORF36 probe F** | GCGGCCGCGCTACCCGGATTTCAGAGAGAC |
| **ORF36 probe R** | GAATTCTAACACTGGAAGAGGACGCAAGA |
| **Hp7** | GAATTCGGGGCGCGTGGTGGCGGCTGCAGCCGCCACCACGCGCCCCGGTACC |
| **ORF72 F** | GGGCCCGAATTCATGGCAACTGCCAATAACCC |
| **ORF72 R** | GGGCCCGAATTCGGTGCCGGCTTGTATATGTGA |
| **ORF34-36 F – Dual Luciferase** | GCGAATTCGGGACAGTGTCGCGTGAATGT |
| **ORF34-36 R – Dual Luciferase** | GAATTCTCAGAAAACAAGTCCGCGGG |
| **ORF35-36 F – Dual Luciferase** | GGGCCCGAATTCATGGACTCAACCAACTCTAAAAGAG |
| **ORF35-36 R – Dual Luciferase** | GAATTCTCAGAAAACAAGTCCGCGGG |
| **ORF35 F – Dual Luciferase** | GGGCCCGAATTCATGGACTCAACCAACTCTAAAAGAG |
| **ORF35 R – Dual Luciferase** | GGGCCCGAATTCTAGGGAGTTTCAGGGCACA |
| **ORF35 R Δ96 R** | GGGCCCGCGGCCGCTTAATTCTCTTCCAGCGCATCTAGG |
| **HA-GFP F** | GGGCCCGCTAGCATGGCTTACCCATACGATGTACCTGACTATGCGG TGAGCAAGGGCGAGGAGCT |
| **HA-GFP R** | GGGCCCTCTAGATTACTTGTACAGCTCGTCCATGC |
| **BAC16 Δ2 F** | TATCTAATCATCCCATCGTATGACATACCGGCGATCATCACCTTGATCAAGGAGAATGGACTCAAAGGATGACGACGATAAGTAGGG |
| **BAC16 Δ2 R** | TAAACTCTCTTTTAGAGTTGGTTGAGTCCATTCTCCTTGATCAAGGTGATGATCGCCGGTATGTCAAACCAATTAACCAATTCTGATTAG |

*F: Forward

*R: Reverse
